# Food Safety Controls during Bulk Food Preparation—An Observational Analysis

**DOI:** 10.3390/foods12122376

**Published:** 2023-06-15

**Authors:** Sri Harminda Pahm Hartantyo, Renuka Selvaraj, Jiaying Ho, Jia Quan Oh, Jun Cheng Er, Angela Li, Kyaw Thu Aung

**Affiliations:** 1 National Centre for Food Science, Singapore Food Agency, 7 International Business Park, Singapore 609919, Singapore; 2 School of Biological Sciences, Nanyang Technological University, 60 Nanyang Drive, Singapore 637551, Singapore

**Keywords:** catering, bulk food preparation, food safety, food hygiene, food safety policy

## Abstract

Seeing the palpable impact of food poisoning associated with catering operations, we surveyed caterers with and without past hygiene violations to examine staffing, food safety practices and correlations with microbial counts in food and the processing environment. Past infringements did not negatively impact the current execution of food safety measures nor the microbial quality of food. In preference to added stringencies for errant operators, we discuss alternative efforts to augment food safety, as well as the policy implications thereof.

## 1. Introduction

Commercial catering premises are frequently identified as settings of foodborne disease outbreaks [1]. Foodborne illness associated with caterers could affect numerous consumers in a much larger proportion than with any other type of establishment in the food service sector [2]. A 2018 report from the Centers for Disease Control and Prevention noted that caterers accounted for more than 20% of foodborne illnesses in the United States, even though less than 15% of reported outbreaks were linked to these catering services [3]. In Singapore, at least 30% (1022/3165) of food poisoning notifications in 2015–2018 were associated with licensed caterers, whereby the majority of cases developed gastroenteritis symptoms after consuming catered food [4].

To the best of our knowledge, data on the causative agents, contamination events or hygiene non-conformities leading to food poisoning at catering sites are limited, and epidemiological investigations following such incidences may be challenged by the lack of contemporaneous information to establish links between contamination sources and affected cases. However, studies have reported food safety concerns in catering services to include cross-contamination due to bulk food preparation, food hygiene non-conformities by caterers taking orders beyond capacity and time-temperature abuse due to prolonged storage of ready-to-eat (RTE) dishes at improper temperatures [1,5,6].

Noting the reported risk factors and with a general guiding principle to identify practices within a company’s activities that are critical to food safety, caterers’ manpower resources and hygiene control practices during bulk food preparation were surveyed through interviews and on-site observations. The study aimed to elucidate whether hygiene non-conformities and poor microbiological counts could be associated with the allocation of manpower resources (and its proportion to scale of operations); or with the practice or omittance of certain hygiene measures. The rationale for stratifying caterers according to outcomes of past hygiene inspections was based on the objective to explore whether there could be notable differences in the way caterers with and without prior hygiene non-conformities implemented food safety procedures.

Gathering such food safety insights on catering businesses is important for awareness of how food safety measures including good personal and food hygiene practices are implemented among catering establishments. Such awareness could help authorities and industries to inform food safety policies and strengthen food safety practices towards risk mitigation.

## 2. Methods

### 2.1. Selection of Study Participants

Via stratified random sampling, we selected participants for this descriptive analysis—10 caterers with at least 1 hygiene-related non-conformity found during routine hygiene inspections, and 10 caterers without.

To select potential study participants, a list of inspection records conducted on caterers within a 2-year period (2017–2018) was generated; each caterer in this list was then assigned with a unique identifier. The Microsoft Excel randomization function was applied and subsequently used to randomly select 10 unique identifiers with and without history of hygiene non-conformity. The caterers corresponding to these unique identifiers were contacted and verbal permission was sought, prior to the study team’s visit to the catering premise for collection of data and samples.

### 2.2. Interviews on Manpower and Business Practices Affecting Food Safety

To gain insight on business practices that may impact food safety, data on the caterers’ typical scale of operations, corresponding staff strength and on the management of increased orders during peak seasons were collected through a researcher-administered questionnaire. One food safety manager, senior-level staff or the business owner themself was interviewed at each premise.

### 2.3. Observations on the Implementation of Food Hygiene Measures

Time-temperature and contamination controls as well as food handler hygiene practices were assessed using an observational checklist developed with reference to the Singapore Environmental Public Health (Food Hygiene) Regulations [7] and on guidelines and educational materials for caterers from the Australia Food Authority [8], Hong Kong Centre for Food Safety [9] as well as the United States Food and Drug Administration [10]. Pre-defined terms used in the checklist (e.g., “sanitary condition” to mean physically clean and orderly) were drawn from these resources to ensure consistency of observations. To ensure objectivity, observations were conducted by two researchers at a time; during which, researchers were unaware of whether the caterers being assessed had prior food hygiene non-conformities.

### 2.4. Collection of Food and Environmental Samples for Microbiological Testing

A total of 86 samples of food and 120 swabs of direct food contact surfaces (e.g., cooking utensils, chopping boards, food containers) and surfaces in close proximity to food (e.g., chiller/freezer/door handles, door exit/entry buttons) were collected for the microbiological analysis. At each catering site, ~250 g of each sample from up to 6 types of dishes designated for delivery on the same day were aseptically collected and kept in sterile bags. Sterile wooden cotton-tipped applicators were used to swab food contact surfaces; these were suspended in 9 mL of Butterfield’s phosphate buffer. Both food and swab samples were then stored in cooler bags and transported to a commercial testing laboratory and refrigerated immediately (~4 °C) until the time of testing.

As a proxy measure for growth of viable bacteria, the laboratory determined aerobic plate counts of the samples (FDA BAM Online, Chapter 3). This was done to determine potential sources of cross-contamination during mass production of food, and to establish correlations between microbial findings and the implementation of food hygiene controls.

### 2.5. Statistical Analysis

Statistical analyses were performed using IBM SPSS Statistics version 26. Kruskal–Wallis test was used to compare the microbiological profiles across different food samples (dishes) and swabs (food contact surfaces). A *p*-value of <0.05 was considered significant.

## 3. Results and Discussion

### 3.1. Scale of Operation Alone Did Not Determine the Occurrence of Hygiene Non-Conformities

Caterers’ scale of operation and manpower resources are summarized in Table 1. Participating caterers served between 80–3000 (median 800) meals per day. In terms of staff strength, the daily number of meals prepared per person ranged from 20 to 500 during peak season (median 100). Caterers without past offences tend to prepare ~30% fewer meals per day (1:70 ratio of staff to median daily orders) than premises with prior hygiene non-conformities (1:100 staff). Between catering sites that produced daily orders at/below the median and those above it, hygiene practices were not observed to be notably different; microbial counts of food processing surfaces likewise did not differ significantly (Mann–Whitney, *p* > 0.05). While it could be expected that a lower volume of orders would allow for more careful attention to food safety, our results instead showed that caterers preparing daily orders at/below median had higher Aerobic Plate Counts (APC, *p* < 0.05) in food samples compared to caterers with daily orders exceeding the median. As the association between caterers’ past record of hygiene violation and scale of operation could not be established, it appears that other factors such as staff strength and the company food safety culture could play a role in the occurrence of hygiene infringements and the execution of food safety measures when preparing food in bulk.

### 3.2. A Food Safety-Oriented Culture Calling for Consistent Ownership of Food Safety Habits across Catering Operations Is Essential to Ensure Safe Food, Especially When Order Quantities Peak

During local festive periods when orders would surge, caterers reported preparing up to five times the number of meals normally made. During these peak seasons, however, 40% of respondents informed us that no adjustments are made despite the increase in workload, while some caterers coped by increasing temporary hires, adopting automation, outsourcing certain food preparations, purchasing RTE food or by limiting orders (Table 1). Without staffing adjustments, more time would be needed to prepare larger orders, leading to a longer time gap between preparation and consumption. The greater workload would also require staff to work faster to prepare bigger quantities of food, inadvertently increasing the risks of improper time-temperature control, cross-contamination, and related hygiene non-conformities. In addition, to facilitate food preparation, several caterers buy pre-prepared RTE food, which would then be stored or further handled (sliced, packed or assembled) to prepare the final dishes. Those without hygiene non-conformities reported keeping these pre-prepared RTE ingredients for a shorter period (<1 day) before serving, as compared to those with prior hygiene non-conformities (up to 3 days). While all caterers filed invoices of these RTE ingredients for record-keeping, none checked the quality nor linked batches of RTE supplies to the dishes they prepared. Should food safety incidences occur downstream, it would hence be difficult to associate whether contamination was due to errant practices during food handling, or due to their use of inherently contaminated RTE ingredients.

The above scenarios highlight the importance of having a food safety-oriented company culture, where value is placed on the formation and ownership of positive food safety habits by all members of the organization, at each stage of the catering operations. This begins with business owners and managers who recognize the significance and are committed to ensuring the safety and traceability of food produced in their facilities [11]. A food safety culture also involves the management’s commitment to developing capable managers who can effectively coordinate food preparation and staff schedules; who have the aptitude to rationally gauge resources needed to prepare food in bulk; and the authority to establish or enact company guidelines for declining orders when they exceed the available resources. Indeed, company leadership that is active and resolute towards training staff to effectively track and manage the interplay between food supplies, manpower and food processing facilities, is vital to preventing incidences that can compromise food safety [12].

### 3.3. Risk Scores Related to Temperature Control, Cross-Contamination Control and Food Handler Hygiene Did Not Significantly Differ (Kruskal–Wallis, p > 0.05) between Premises with and without Previous Hygiene Non-Conformities

Food hygiene practices observed across catering sites are given in Table 2. For temperature control, all caterers had working freezers/refrigerators solely for raw ingredients, potentially higher-risk ingredients (i.e., those requiring time-temperature control) were generally handled well, and nearly all caterers were observed to maintain these ingredients on ice or at ≤0 °C to 4 °C upon receiving, until the time of preparation. However, the time gap between delivery of the ingredients by suppliers and its receipt/storage by caterers could not be practicably observed and is thus not known. Other temperature-control-related inadequacies were noted—frozen food was thawed at ambient temperature (65%, 11/17); large servings of hot RTE food were cooled at ambient temperature (36%, 5/14) and holding temperatures were not maintained after preparation (76%, 13/17) and subsequent transportation to customers or other business units. The deviations in cold chain management of temperature-sensitive ingredients, the unsafe thawing and the lack of temperature control for RTE food may lead to increased core temperatures and subsequent bacterial proliferation in food. For raw RTE foods that do not undergo heat treatment, initial microbial loads can be relatively higher, and thus keeping temperatures outside the danger zone (5–60 °C) is crucial to prevent the proliferation of microbes, especially those that are pathogenic, to harmful levels.

In terms of cross-contamination controls—the majority of caterers (≥80%) practiced the segregation of raw and RTE food throughout its operations, but soiled premises (e.g., scraps of food littered on the floor and preparation areas) and food contact surfaces (e.g., visible food residues) were observed when there was no on-going food preparation activities both at premises with and without prior hygiene non-conformities (25%, 5/20), see Table 2. The use of common chopping boards for raw and cooked food (21%, 4/19) were seen; unsanitary environmental conditions such as dirty pipes and stagnant water puddles in close proximity to food preparation areas (25%, 5/20) were also noted. The majority of food handlers had good personal hygiene (85%, 17/20) and are prohibited from handling food when sick (84%, 16/19), but inadequate hand-washing facilities were observed in 45% (9/20) of catering kitchens. These inadequacies included one or a combination of the lack of soap, the lack of hand-drying implements such as hand towels or dryers and the inaccessibility of handwashing facilities in food preparation areas. The cruciality of handwashing with soap is emphasized in numerous studies; contact time and mechanical actions when washing with soap result in more effective removal of bacteria than washing with water alone [13,14]. On the same note, washing without drying poses a food safety risk as transmission of bacteria is more likely when skin is wet. Proper laundering of non-disposable hand towels is also important to ensure food safety; a substantially higher prevalence of foodborne bacteria has been reported on hands dried with reusable dishcloths as compared to hands dried with single-use paper towels [15].

### 3.4. Poor Microbiological Profiles Were Associated with Dishes Containing Ingredients with High Inherent Bacterial Loads

**Figure 1:**
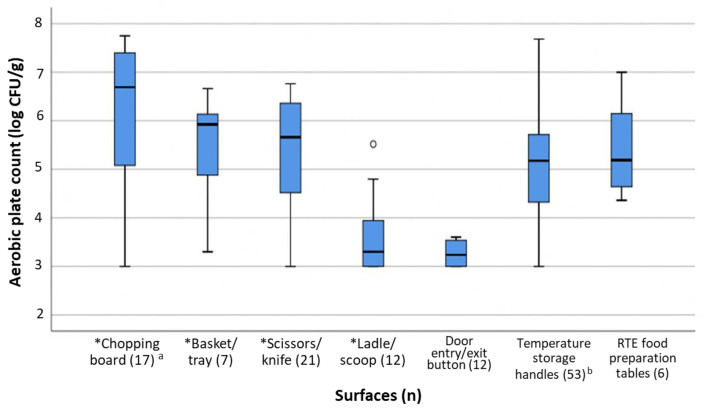
Microbiological quality of food processing surfaces in catering establishments. Surfaces or utensils with asterisks (*) refer to direct food-contact surfaces (Zone 1); those without indicate surfaces in close proximity to food preparation areas (Zone 2). Superscript a indicates significantly higher bacterial counts (*p* < 0.05) than other surfaces in the same Zone; b includes door handles of warmers, chillers, refrigerators and freezers. Symbol “o” in the figure refers to an outlying result.

About 10% (9/86) of RTE food collected from catering establishments had poor microbiological profile (as approximated by APC exceeding 5 log CFU/g). The median APC of all food samples collected was ~2 log colony forming units per gram (CFU/g), and no significant differences (*p*-value > 0.05) in microbiological profiles were observed between RTE food samples collected from premises with and without prior hygiene non-conformities. Similarly, no significant associations could be established between microbiological profile and the implementation (or lack) of hygiene controls in premises where they were prepared. Rather, poor microbiological profiles were observed to be correlated to certain food types, whereby dishes consumed raw or containing raw ingredients (such as salads and sandwiches); and dishes subjected to heavy post-cooking manipulation such as desserts accounted for the ~10% of food with high APCs. Noting the differences in the natural microflora in food, dishes that contain ingredients with inherently higher APCs (such as raw vegetables) should therefore be handled with extra precaution, to avoid further contamination of the ingredient, or cross-contamination of other food items. Figure 1 shows total bacteria counts of 120 swabs collected across direct food contact surfaces (Zone 1, n = 57) and surfaces in close proximity to food contact surfaces (Zone 2, n = 63). No significant difference was observed among microbial counts of these surfaces in premises with and without a history of hygiene non-conformities (*p*-value > 0.05). Chopping boards tended to have significantly higher bacterial counts (*p*-value < 0.05) as compared to other Zone 1 food contact surfaces such as knives/slicers, food trays, and ladles as well as Zone 2 surfaces such as preparation tables, doorknobs and entrance/exit buttons. This finding is not surprising; a substantial bacterial load can remain on boards with hard-to-clean cracks or crevices from prolonged use and can persist even with frequent rinsing before and after every use [16]. Bacteria on these chopping boards can transfer onto other food items, progressively contaminating other direct food contact surfaces and contributing to microbial counts on RTE food, especially when segregation of utensils for raw and uncooked food is not practiced. In some cases of foodborne illness, the point of contamination leading to food poisoning has been traced back to kitchen surfaces such as chopping boards where implicated food items were prepared [17,18].

### 3.5. Implications to Food Safety Guidance and Policy

National food safety laws such as the Food Safety Modernization Act in the US [19], the General Food Law [20] in Europe, the Food Safety Basic Act in Japan [21] and the Food Hygiene Regulations in Singapore [7] mandate food business licensees to have systems that ensure the safety of food used in and/or supplied by their establishments. These international food policies emphasize the need for practices that prevent foodborne hazards, ahead of response systems to manage food safety breaches after they occur. Food safety laws are generally outcome-based, where establishments are ultimately required to produce and prepare food that is safe for human consumption; broadly speaking, this goal can be achieved through prescriptive means (i.e., requiring food operators to comply with well-defined food hygiene standards set out by local authorities) or through self-regulation by the food business. More recently, a mixture of these approaches has surfaced as an “enforced” self-regulation where food businesses are required to identify risks specific to their operations and thereafter form and comply with a food safety program to control these contingencies [22]. Regardless of the approach taken, food safety compliance is then typically monitored through routine on-site inspections or advisory visits; the purpose and schedule of appointments are set forth by regulatory agencies and frequencies can differ based on the type of business, on previous records or as a follow-up to complaints [23]. Prescriptive interventions, however, may be seen as a limitation in terms of its applicability and practicability to food operations, and such interventions may not always guarantee a positive food safety outcome, as seen in our study where hygiene non-conformities were observed even in premises that did not give rise to the finding of food safety lapses during the past cross-sectional inspections. These limitations are further alluded to by studies that report varying associations between interventions and longer-term outcomes—Allwood et al. [24] noted a decline in inspection ratings when the visit frequency decreased; Kosola et al. [25] found that inspection outcomes are affected by whether visits were pre-announced and by how food establishments perceive the level of food safety risk in their operations; while Jones et al. [26] was unable to observe significant differences in compliance ratings based on the time since the previous inspection.

Our analyses potentially benefit food safety policy implications, as it showed that a caterer’s record of past hygiene non-conformities, or the lack thereof, did not significantly influence their current implementation of food hygiene controls nor the microbiological quality of the food prepared in their premises. As opposed (or in addition) to closer monitoring of errant operators, it may thus be more fruitful to focus efforts on ensuring the safe preparation of higher risk food items, as well as on staff training. In Singapore, as in other nations [27,28,29], food handlers are required to complete a basic food safety course or expected to have supervised food safety training, prior to their registration and/or subsequent employment in food service establishments. Food hygiene resources and educational materials are publicly accessible, available in major local dialects, and cover a wide range of topics such as personal hygiene, food storage, upkeep of premises and the like. Nevertheless, a company committed to cultivating a food safety culture may develop its own set of supplemental training resources that focus not only on sanitary food preparation, but on how to effectively and efficiently maintain these hygienic practices when handling significantly larger volumes of food; studies have shown that training plans are more effectual when drawn from specific problems and clearly defined goals within the framework and context of its operations [30,31].

To further contextualize food safety training for catering operations, module content may also mention the adoption of practices such as those noted in this study—the use of automation to cope with larger orders, the use of blast chillers to rapidly cool cooked food and the use of thermometers to ensure thorough cooking—as well as the clear articulation of how each food handler’s habits can impact the entirety of an order and the wellbeing of multiple consumers. Organizations can further facilitate a food safety culture by providing ancillary training for staff to upskill in terms of numeracy (to coordinate supplies, manpower, time and physical resources to efficiently prepare large quantities of food within a reasonable time frame); in risk assessment (to systematically troubleshoot and mitigate food safety hazards); as well as in book-keeping (to facilitate traceability of RTE ingredients and prepared dishes, especially if premises serve as a central kitchens for multiple retail outlets). With ancillary training, management may also consider providing subsequent support and reward structures to drive the application of concepts learned; a study on the attitudes of industrial caterers towards training programs indicated that follow-up and support from management fosters a positive attitude and motivation among employees to apply learnings in daily operations [30]. Such program structures could also benefit establishments that cater to more vulnerable populations such as collective homes for the elderly or childcare centers, where added attention is needed to consistently ensure safe food for the immunocompromised.

Notably, the majority of catered foods sampled for this study had total bacteria loads below 5 log CFU/g, affirming sound efforts by the industry and by local authorities in safeguarding catered food. However, given the heterogeneity and volume of food prepared at catering establishments, a careful analysis of the product’s characteristics and the process conditions required to minimize associated hazards would be worthwhile, whereby the preparation of food types more prone to spoilage, undercooking and higher microbial loads (e.g., raw RTE dishes, thick slabs of meat, dairy products, dishes that require assembly or the incorporation of pre-prepared ingredients) are afforded as much attention allowable, without compromising the microbiological safety of lower-risk food types (e.g., deep-fried dishes). A similar risk-based approach may also be adopted during inspections such that the preparation of higher-risk foods or food operations catering to vulnerable communities be more closely observed and immediate guidance be provided, should food safety measures be found lacking, or should reluctance to practice safe measures be perceived among food handlers. It has been shown that a food safety regimen guided by food type, process controls and intended population could mitigate or reduce food-safety risks [32,33].

## 4. Conclusions

Lastly, in highly industrialized places such as Singapore where space is limited and manpower is costly, the commitment of food business operators to instill food safety culture, the competence of workers to hygienically handle food, and the ability of food service managers to effectively coordinate resources and leverage on automation are vital to ensuring the safe bulk preparation of food. Likewise, implementing effective controls at points critical to catered food (such as maintaining food-holding temperatures from kitchen to consumer) and having easy-to-follow education modules that emphasize safe mass production of food and encourage a wider adoption of best practices (e.g., the use of blast freezers to rapidly cool cooked food; maintaining the cleanliness of direct- and high-touch food contact surfaces) could further strengthen the catering industry’s acumen against potential food safety risks. Although our study could have benefitted from a larger sample size, it shows that when large amounts of food need to be prepared in a short amount of time, prioritizing vigilance on high-risk food types and on key processing areas are imperative to minimize downstream incidences of foodborne illness and to provide consumers with safe and wholesome food. Nevertheless, our findings put forth important considerations for the formulation, review and further strengthening of food safety policies.

## Figures and Tables

**Table 1:** Respondents’ scale of operations and staff strength.

Type of Catering Premise (*n*)	Median (Lower–Upper Limits)			Adjustments Made during Peak Periods
	Daily Meal Orders per Premise ^1^	Staff Strength per Premise	Meals Prepared per Staff per Day	
Caterers with history of hygiene non-conformities (10)	800 (27–3000)	10 (4–30)	100 (31–200)	No adjustment (3), hire extra help (2), automation (1), purchase RTE food (1), stop taking orders (1), Don’t know (2)
Caterers without history of hygiene non-conformities (10)	900 (80–2000)	8.5 (4–25)	70 (20–500)	No adjustment (5), hire extra help (2), purchase RTE food (1), Don’t know (2)
	800 (27–3000)	9 (4–30)	100 (20–500)	

^1^ Upper limit value refers to maximum number of orders during peak periods.

**Table 2:** Food hygiene practices observed at catering premises.

Food Hygiene Control Parameters	Caterers Implementing Food Hygiene Controls, % (n) ^1^		
	With Prior Hygiene Non-Conformities ^6^	Without Prior Hygiene Non-Conformities	Total
Temperature controls ^2^			
Designated refrigerator/freezer used for raw food	100 (10/10)	100 (10/10)	100 (20/20)
Designated refrigerator/freezer used for cooked/RTE food	60 (6/10)	60 (6/10)	60 (12/20)
Temperature of frozen/chilled food maintained from receiving to pre-preparation	100 (9/9)	89 (8/9)	94 (17/18)
Thawing of food is not done at ambient temperature	38 (3/8)	33 (3/9)	35 (6/17)
Cooling of cooked food is not done at ambient temperature	50 (3/6)	75 (6/8)	64 (9/14)
RTE food temperature controlled after cooking/preparation	33 (3/9)	12 (1/8)	24 (4/17)
RTE food temperature controlled during transport to catering site	100 (2/2)	50 (1/2)	75 (3/4)
Cross-contamination/sanitation controls			
Premises are kept in sanitary condition ^3^	50 (5/10)	100 (10/10)	75 (15/20)
Food contact surfaces are in sanitary condition	70 (7/10)	80 (8/10)	75 (15/20)
Proper disposal measures ^4^ are in place	80 (8/10)	100 (10/10)	90 (18/20)
Signs of pest infestation observed	40 (4/10)	60 (6/10)	50 (10/20)
Raw and RTE food segregated upon receiving	100 (9/9)	57 (4/7)	81 (13/16)
Raw and RTE food segregated at storage, before preparation	89 (9/10)	89 (8/9)	90 (17/19)
Raw and RTE food segregated during preparation	67 (6/10)	78 (7/9)	94 (17/18)
Raw and RTE food segregated after preparation	90 (9/10)	67 (6/9)	80 (15/19)
Designated chopping boards used for raw and RTE food	80 (8/10)	100 (8/8)	80 (15/19)
Designated knives used for raw and RTE food	80 (8/10)	88 (7/8)	83 (15/18)
Food handler (FH) hygiene			
FHs avoid unnecessary contact with RTE food and food contact surfaces	44 (4/9)	78 (7/9)	61 (11/18)
Hand washing facilities (water basin, soap, hand towels) are available	50 (5/10)	60 (6/10)	55 (11/20)
Hand washing facilities are used exclusively for hand washing	56 (5/9)	80 (8/10)	68 (13/19)
FHs wash and dry hands thoroughly	50 (4/8)	60 (6/10)	56 (10/18)
FHs wash hands upon contamination	50 (4/8)	56 (5/9)	53 (9/17)
FHs maintain good personal hygiene ^5^	70 (7/10)	100 (10/10)	85 (17/20)
FHs are prohibited from handling food when sick	78 (7/9)	90 (9/10)	84 (16/19)

^1^ For n < 20, practices were not on-going and could not be observed/assessed at the time of study visit. ^2^ Temperature control at transport—hot food kept in food warmers, chilled food on ice. ^3^ Sanitary condition—physically clean, orderly. ^4^ Proper disposal measures—designated trash/waste area situated away from food preparation area. ^5^ Good personal hygiene—visibly clean hands; clean, tidy attire; hair/beard is neat, tidy and tied or restrained if long. ^6^ Refers to infringements under the Environmental Public Health (Food Hygiene) Regulations, based on 2016–2017 inspection records.

## Data Availability

The data presented in this study are available on request from the corresponding author. The data are not publicly available, to maintain privacy of respondents.
